# *GPR174* and *ITM2A* Gene Polymorphisms rs3827440 and rs5912838 on the X chromosome in Korean Children with Autoimmune Thyroid Disease

**DOI:** 10.3390/genes11080858

**Published:** 2020-07-27

**Authors:** Won Kyoung Cho, Hye-Ri Shin, Na Yeong Lee, Seul Ki Kim, Moon Bae Ahn, In-Cheol Baek, Tai-Gyu Kim, Byung-Kyu Suh

**Affiliations:** 1 Department of Pediatrics, College of Medicine, St. Vincent’s Hospital, The Catholic University of Korea, Seoul 065941, Korea; wendy626@catholic.ac.kr; 2 Catholic Hematopoietic Stem Cell Bank, College of Medicine, The Catholic University of Korea, Seoul 065941, Korea; hyeris@catholic.ac.kr (H.-R.S.); icbaek@catholic.ac.kr (I.-C.B.); 3 Department of Pediatrics, College of Medicine, Seoul St. Mary’s Hospital, The Catholic University of Korea, Seoul 065941, Korea; ibboon2@catholic.ac.kr (N.Y.L.); seulki12633@gmail.com (S.K.K.); mbahn@catholic.ac.kr (M.B.A.); 4 Department of Microbiology, College of Medicine, The Catholic University of Korea, Seoul 065941, Korea

**Keywords:** autoimmune thyroid disease, gender, *GPR174* and *ITM2A*

## Abstract

(1) Background: Autoimmune thyroid diseases (AITDs) are female predominant and much attention has been focused on G protein-coupled receptor 174 (*GPR174)* and integral membrane protein 2A (*ITM2A)* on the X chromosome as Grave’s disease (GD) susceptible locus. (2) Methods: We genotyped four single nucleotide polymorphisms (SNPs), rs3810712, rs3810711, rs3827440, and rs5912838, of *GPR174* and *ITM2A* in 115 Korean children with AITD (M = 25 and F = 90; GD = 74 (14.7 ± 3.6 years), HD = 41 (13.4 ± 3.2 years); GD-thyroid-associated ophthalmopathy (TAO) = 40, GD-non-TAO=34) and 204 healthy Korean individuals (M = 104 and F = 100). The data were analyzed by sex-stratified or combined. (3) Results: Three SNPs, rs3810712, rs3810711 and rs3827440, were found to be in perfect linkage disequilibrium (*D*’ = 1, r^2^ = 1). In AITD, HD, GD, GD-TAO, and GD-non-TAO patients, rs3827440 TT/T and rs5912838 AA/A were susceptible and rs3827440 CC/C and rs5912838 CC/C were protective genotypes. When analyzed by sex, rs3827440 TT and rs5912838 AA were susceptible and rs3827440 CC and rs5912838 CC were protective genotypes in female AITD, GD, GD-TAO, and GD-non-TAO subjects. In male AITD patients, rs3827440 T and rs5912838 A were susceptible and rs3827440 C and rs5912838 C were protective genotypes. (4) Conclusions: Polymorphisms in *GPR174* and *ITM2A* genes on the X chromosome might be associated with AITD in Korean children.

## 1. Introduction

Autoimmune thyroid disease (AITD) may occur when genetically susceptible individuals are exposed to environmental triggers such as infection, iodine, or stress [1]. The genetic factor has a major role in AITD etiology and the heritability of Graves’ disease (GD) has been reported to be 79% [2]. AITD encompasses GD and Hashimoto’s thyroiditis (HD) [3]. HD seems to involve a CD4 Th1 response. The effects of antibodies and effector T cells specific for thyroid antigens lead to the progressive destruction of normal thyroid tissue. The autoimmune response in GD is biased towards a CD4 Th2 response and is focused on antibody production. The production of anti-thyroid Stimulating Hormone (TSH) receptor antibodies promotes chronic overproduction of thyroid hormone [3]. However, the fact that GD and HD are commonly observed in the same family tree reflects a similar genetic basis for these diseases [4,5]. In early onset autoimmune disease, genetic susceptibility might be greater concern than in late onset cases [6]. We have reported an increase in allele frequencies of HLA-B*46, -DRB1*08, and -Cw*01 [7]. The statistical significance in our previous study was much higher than observed in other studies conducted on Korean adults [8], which might suggest that early-onset AITD is more influenced by genetic factors than late-onset AITD.

The female predominance in autoimmune disease has long been recognized and the most striking sex differences in prevalence are observed in AITD (>80% women) [9,10]. However, the biology of sexual dimorphism in AITD is not clearly understood. Recently, a great deal of attention has focused on sexual dimorphism in the immune response. Sex dimorphisms in immune response appear to be partially associated with direct genetic differences such as X chromosome and linked genes, and sex hormone and sex-specific regulation of immune-related genes [11]. The X chromosome contains approximately 1000 genes, including many immune-related genes that encode receptors and associated proteins, immune response-related proteins and transcriptional and translational regulator [12]. Some genes located on the X chromosome may play an important role in susceptibility to GD [13]. Furthermore, X chromosome inactivation and skewing might be important contributors to the increased risk for AITD in females [14,15,16].

**Figure 1:**
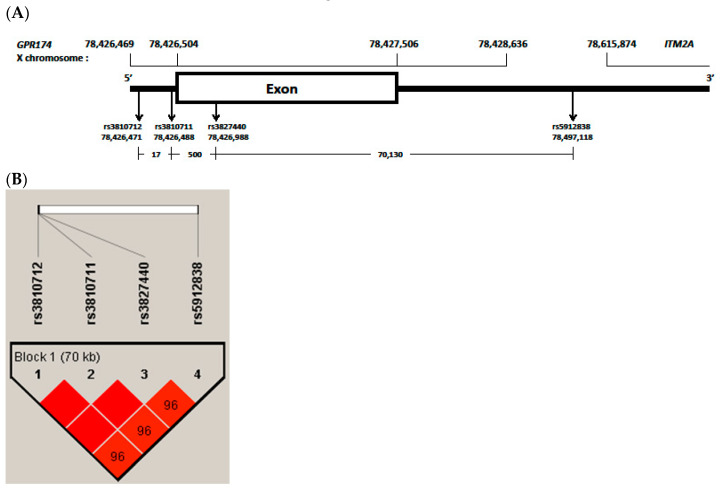
(**A**), G protein-coupled receptor 174 (*GPR174)* and integral membrane protein 2A (*ITM2A)* variants region have been reported from GRCh37.p13 (hg19). rs3810712, 5 prime untranslated region (‘UTR) variant, C>G,T; rs3810711, 5′UTR variant, T>C,G; rs3827440, missense variant, T>A,C,G; rs5912838, 3′UTR variant, A>C,G; (**B**), block and frequency of linkage disequilibrium (LD) of selected four single nucleotide polymorphisms (SNPs) in *GPR174*. Boxes are colored deep red if the D’ values are high, which means LD is more strong. Especially, rs3810712, rs3810711 and rs3827440 were found to be in perfect linkage disequilibrium (*D*’ = 1, r^2^ = 1).

Previous studies found associations with genes on the X chromosome in patients with AITD. Associations of AITD have been reported with polymorphisms of FOXP3 in Caucasian [17] and Japanese [18], TLR7 in a Chinese Cantonese population [19] and IRAK1 in Chinese [20]. We also reported that polymorphisms of IRAK1 gene on X chromosome is associated with HD in Korean children [21]. Recently, the rs3827440 and rs5912838 single nucleotide polymorphisms (SNPs) in G protein-coupled receptor 174 (*GPR174*) and integral membrane protein 2A (*ITM2A*) on the X chromosome were suggested to be GD-susceptible loci after the major histocompatibility complex region [13,22]. However, to the best of our knowledge, there have been no reports on possible associations of *GPR174* and *ITM2A* polymorphisms with AITD in Korean children. In this study, we investigated the role of *GPR174* and *ITM2A* polymorphisms (rs3810712, rs3810711, rs3827440, rs5912838) with AITD in Korean children (Figure 1A).

## 2. Materials and Methods

### 2.1. Subjects

The present study is a noninterventional registry study. There were 206 patients diagnosed with AITD who agreed to participate in this study between March 2009 and August 2019 in the pediatric endocrine clinic at Seoul St. Mary’s and St. Vincent’s Hospitals. Among these 206, subjects who had blood sample insufficient for genetic study (n = 91) were excluded. Ultimately, our study included 115 patients (90 females and 25 males) diagnosed with AITD (41 HD and 74 GD cases). The mean age (±SD) of GD patients at enrollment was 14.7 ± 3.6 years and HD patients was 13.4 ± 3.2 years. Among the 74 GD patients, 40 patients had thyroid associated ophthalmopathy (TAO) (Table 1).

For the control group, 204 healthy and genetically unrelated Korean adults (100 females, 104 males) without a history of AITD were included. The control group was mainly comprised of students and staff from the Medical College of the Catholic University of Korea and hematopoietic stem cell transplantation (HSCT) center. In general, the health status of students is considered to be free of special problems. All subjects provided informed consent to participate in a genetic study. The Institutional Review Board of the Catholic University of Korea approved our study (IRB Number: KC09FISI0042, MC13SISI0126).

HD was diagnosed when at least three of Fisher’s criteria [26] were met: (1) goiter, (2) diffuse goiter and decreased uptake at thyroid scan, (3) the presence of circulating thyroglobulin and/or microsomal autoantibodies, and (4) hormonal evidence of hypothyroidism. GD diagnosis was based on confirmation of clinical symptoms and the biochemical confirmation of hyperthyroidism, including the observation of goiter, elevated ^131^I uptake by the thyroid gland, positive TSH receptor antibodies and elevated thyroid hormone levels. In GD, a remission was defined as consistent with the improvement of clinical features and restoration of euthyroidism or induction of hypothyroidism after ATD therapy. We defined an intractable as hyperthyroidism persistent over 2 years of ATD therapy or relapsed after ATD withdrawal or had been treated ATD for at least 5 years [23,24,25]. Patients who had other autoimmune diseases, hematologic diseases and endocrine diseases were excluded. TAO was diagnosed based on the presence of typical clinical features and classified according to the system recommended by the American Thyroid Association Committee. Patients with no symptoms or only lid lag sign were included in the without-TAO group [27]. Patients with soft tissue changes, proptosis, extraocular muscle dysfunction, or the latter two symptoms, were considered to have TAO [28].

### 2.2. DNA Extraction

Genomic DNA was extracted from 4 mL of peripheral blood mixed with ethylenediaminetetraacetic acid using TIANamp Genomic DNA Extraction Kits (Tiangen Biotech Corporation, Beijing, China), according to the manufacturer’s instructions. The concentration of the DNA solution was adjusted to 100 ng/μL, and the solution was stored at −20 °C. Samples were used as a polymerase chain reaction (PCR) template for genotyping [29]. 

### 2.3. Target Gene Primer Design and Multiplex PCR

Four primers designed for *GPR174* (rs3810712: C>T), *GPR174* (rs3810711: T>C), *GPR174* (rs3827440: T>C), *GPR174* and *ITM2A* (rs5912838: A>C) are listed in Table 2. Genomic DNA was acquired from a variety of samples, and AITD pediatric patient and control groups were amenable to analysis using a 50 ng PCR template, or less. The fist amplicons were made by a multiplex PCR process using a Multiplex kit (Cat: 206143; Qiagen, Hilden, Germany) with other genes. In the first PCR, the extracted genomic DNA was amplified in a ProFlex 96-Well PCR System (Thermo Fisher Scientific Waltham, MA, USA) using the following PCR conditions: 1 cycle at 95 °C for 15 min and 40 cycles of denaturation at 94 °C for 30 s, annealing at 63 °C for 90 s, and extension at 72 °C for 30 s. Final extension was at 60 °C for 30 min. In order to enable MiSeq equipment (Illumina, San Diego, CA, USA) to read nucleotides around the target SNP in the next step [30], the PCR2 was carried out with PCR1 amplicon and Illumina universal primer [31]. The universal primer consists of tag, index and adapter sequences. Using 8 bases for each index, to distinguish each sample of PCR1 amplicon, 8 forward primers and 6 reverse primers were employed as universal primers, resulting in 48 unique combinations for PCR2 (Table 2) [32]. The primer information is provided by Illumina, Inc. for SNP typing. The PCR2 primer amplification was performed in a ProFlex 96-Well PCR System (Thermo Fisher Scientific) using the following conditions: 1 cycle at 95 °C for 15 min and 35 cycles of denaturation at 95 °C for 30 s, annealing at 59 °C for 30 s, and extension at 72 °C for 60 s.

### 2.4. Sequencing

We used Illumina MiSeq equipment for typing of target X chromosome genes. A sample sheet was prepared on the MiSeq sequencer (Illumina) to provide run details [33]. A standard flow-cell was inserted into the flow-cell chamber. The pooled sample was diluted with chilled HT1 (Hybridization Buffer) to a concentration of 2 nM, and an equal amount of 0.2 N NaOH was added to denature the sample; the mixture was incubated for 5 min. A PhiX control sample at 2 nM was denatured in the same way. Both the sample and the PhiX were diluted to 8 pmol/L and 1% PhiX was added to the sample. Then, 600 μL of the spiked sample with a final concentration of 8 pmol/L was pipetted into the sample well on the MiSeq consumable cartridge before loading in the cooling section of the MiSeq machine. Sequencing was performed on a MiSeq sequencer using 151 bp paired-end reads, including an index run according to the manufacturer’s instructions (MiSeq System user guide part #15027617 Rev. C April 2012, MiSeq Reagent kit 300 cycles, Box1 (ref 15026431) and Box2 (ref 15026432)) [34]. To the validation, Sanger sequencing and Integrative Genomics Viewer (IGV) version 2.5.2 were implemented [35].

### 2.5. Data Analysis

Data analysis was performed using the MiSeq output report binary alignment map (BAM) file mapping Burrows-Wheeler Aligner (BWA) Whole Genome Sequencing v1.0. (Illumina) [36]. The proliferation of web-based integrative analysis frameworks has enabled users to perform complex analyses directly through the web using Galaxy [37]. We performed the following steps:

Pre-process Next-generation sequencing (NGS)data:

(1) MAP with BWA and add read group reference: hg19 [36,38]. The sequence alignment/map format (BAM) dataset was preprocessed from the Illumina MiSeq instrument.
(2) The sequence alignment/map format (BAM) dataset was uploaded in the Galaxy website.

Identify variable site:

The sequence alignment/map format (BAM) dataset uploaded in the Galaxy website was used.

(1) NAIVE VARIANT CALLER (Galaxy tool)

Options: restrict analysis to chrX; min number of reads to call variants? (irrelevant); min base quality (BQ) >= 30; min mapping quality (MAPQ) >= 20; ploidy (irrelevant); only write positions with alternate alleles? NO; report counts per strand.

(2) VARIANT ANNOTATOR (Galaxy tool)

Options: parse variant call format (VCF) () to extract counts, major, minor alleles and minor allele frequency (MAF); MAF threshold >= 0%; coverage >= 0; do not filter sites.

(3) Filters

MAF >= 0.25% (in forward and reverse strands); CVRG >= 100x (Total coverage); SB <= 1 (strand bias).

### 2.6. Statistical Analysis

Allele frequencies were determined using Microsoft Office Excel. Fisher’s exact test was applied when the expected frequency was lower than 5. The *p* value was multiplied by the number of alleles observed to give a corrected *p* value (Pc), which accounts for the multiple comparisons performed. A corrected *p* value of <0.05 was considered statistically significant. Haldane’s formula correction was used when critical entries were equal to zero. The data from cases and controls were analyzed by separate sex-stratified or all combined. Hardy–Weinberg equilibrium (HWE) of each SNP in *GPR174* and *ITM2A* were analyzed according to the calculation proposed by Graffelman and Weir [25]. All genotyped SNPs fit the HWE (see Supplementary Tables S1 and S2). To evaluate the presence of linkage disequilibrium (LD) and block of haplotypes between polymorphisms on *GPR174* and *ITM2A*, LD and haplotypes were analyzed using the Haploview software, version 4.2 [39].

Based on *GPR174* and *ITM2A* SNPs MAF (minor allele frequency) of 25% in a dominant model and predicted prevalence of AITD at 2% [40], unmatched case-control design, a total of 315 participants is required to yield statistical power of 80% and type 1 error of 5%. The power of our study was calculated based on an available sample size of 115 cases and 204 controls. The power of MAF 25.0 for rs3810712, rs3810711, rs3827440 and rs5912838 were 0.71–0.83 in the odds ratio (OR) from 1.8 to 2.0. In the OR from 0.2 to 0.5, it was 0.99–0.80. In GD cases, the power was 0.58–0.72 in the OR from 1.8 to 2.0. In the OR of 0.5, it was 0.67. Sample size and power were calculated using Quanto 1.2.4 software (preventivemedicine.usc.edu/ Los Angeles, CA, USA).

## 3. Results

### 3.1. Comparison of Genotype and Allele Frequencies of GPR174 and ITM2A SNPs on the X chromosome in AITD Patients and Controls

All genotyped SNPs fit the HWE (see Supplementary Tables S1 and S2). Three SNPs, rs3810712, rs3810711 and rs3827440, were found to be in perfect linkage disequilibrium (D’ = 1, r^2^ = 1) (Figure 1B). In patients with AITD (n = 115), the genotype and allele frequencies of rs3827440 TT/T (Corrected P (Pc) = 0.019), T (Pc = 0.000), rs5912838 AA/A (Pc = 0.032), and A (Pc = 0.000) were higher and those of rs3827440 CC/C (Pc = 0.000), C (Pc = 0.000), rs5912838 CC/C (Pc = 0.000), and C (Pc = 0.000) were lower than those in controls (n = 204) (Table 3).

In female AITD patients (n = 90), the genotype and allele frequencies of rs3827440 TT (*p* = 0.027), T (Pc = 0.011), rs5912838 AA (*p* = 0.040), and A (Pc = 0.006) were higher and those of rs3827440 CC (*p* = 0.021), C (Pc = 0.011), rs5912838 CC (Pc = 0.017), and C (Pc = 0.006) were lower than those of female controls (n = 100). In male AITD patients (n = 25), the allele frequencies of rs3827440 T (*p* = 0.026), and rs5912838 A (*p* = 0.033) were higher and those of rs3827440 C (Pc = 0.025), and rs5912838 C (Pc = 0.032) were lower than those in male controls (n = 104).

### 3.2. Comparison of Genotype and Allele Frequencies of GPR174 and ITM2A SNPs on X chromosome in GD Patients with or without TAO and Controls

In patients with GD (n = 74), the genotype and allele frequencies of rs3827440 TT/T (Pc = 0.006), T (Pc = 0.000), rs5912838 AA/A (Pc = 0.010) and A (Pc = 0.000) were higher and those of rs3827440 CC/C (Pc = 0.000), C (Pc = 0.000), rs5912838 CC/C (Pc = 0.000), and C (Pc = 0.000) were lower than those of controls (n = 204). In patients with GD-TAO (n = 40), the genotype and allele frequencies of rs3827440 T (Pc = 0.008), and rs5912838 A (Pc = 0.007) were higher and those of rs3827440 CC/C (Pc = 0.008), C (Pc = 0.005), rs5912838 CC/C (Pc = 0.006), and C (Pc = 0.004) were lower than those of controls (n=204). In patients with GD non-TAO (n = 34), the genotype and allele frequencies of rs3827440 TT/T (Pc = 0.009), T (Pc = 0.000), rs5912838 AA/A (Pc = 0.013), and A (Pc = 0.000) were higher and those of rs3827440 CC/C (Pc = 0.002), C (Pc = 0.000), rs5912838 CC/C (Pc = 0.001), and C (Pc = 0.000) were lower than those of controls (n=204).

In female GD patients (n = 52), the genotype and allele frequencies of rs3827440 TT (Pc = 0.022), T (Pc = 0.001), rs5912838 AA (Pc = 0.032), and A (Pc = 0.000) were higher and those of rs3827440 CC (Pc = 0.005), C (Pc = 0.001), and rs5912838 CC (Pc = 0.002), C (Pc = 0.000) were lower than those of female controls (n = 100). In female GD-TAO patients (n = 27), the genotype and allele frequencies of rs3827440 T (Pc = 0.032), and rs5912838 A (Pc = 0.022) were higher and those of rs3827440 CC (*p* = 0.019), C (Pc = 0.032), and rs5912838 CC (Pc = 0.029), C (Pc = 0.022) were lower than those of female controls (n = 100). In female GD-non TAO patients (n = 25), the genotype and allele frequencies of rs3827440 TT (Pc = 0.024), T (Pc = 0.004), rs5912838 AA (Pc = 0.033), and A (Pc = 0.003) were higher and those of rs3827440 CC (*p* = 0.025), C (Pc = 0.004) rs5912838 CC (Pc = 0.041), and C (Pc = 0.003) were lower than those of female controls (n = 100) (Table 4 and Table 5). In female GD remission patients (n = 23), there was no significant difference in rs3827440 TT and rs5912838 AA genotype frequencies compared with intractable groups (n = 51) 

### 3.3. Comparison of Genotype and Allele Frequencies of GPR174 and ITM2A SNPs on X chromosome in HD Patients and Controls

In patients with HD (n = 41), the genotype frequencies of rs3827440 CC/C (Pc = 0.017), and rs5912838 CC/C (Pc = 0.012) were lower than those of controls (n = 204) 

### 3.4. LD and Haplotype Analysis of the Four GPR174 and ITM2A SNPs

All four SNPs, rs3810712, rs3810711, rs3827440 and rs5912838, were in strong LD (*D’* = 0.9–1, r^2^ = 0.92) (Figure 1B). rs3810712, rs3810711, and rs3827440 were found to be in perfect LD (*D’* = 1, r^2^ = 1). The haplotype frequencies of the *GPR174* and *ITM2A* gene in pediatric patients with AITD and controls are shown in Table 6. The haplotype (CTTA) frequencies of *GPR174* and *ITM2A* rs3810712, rs3810711, rs3827440, and rs5912838 were higher in AITD patients than in controls (r^2^ = 20.29, *p* = 0.000).

## 4. Discussion

*GPR174*, which is located in the Xq21.1 region, encodes a protein member of the P2Y receptor family. Lyso-phosphatidyl-serine secreted by the immune system acts as a ligand for *GPR174. GPR174* is extensively expressed in the immune system and thyroid tissue and inhibits the production of the T-helper 1 cytokine [13]. *ITM2A,* which is encoded by a gene also located in the Xq21.1 region, is expressed by CD4+ T cells and plays a role in the activation of T cells [41]. The SNP rs3827440 of *GPR174* is a nucleotide transition in the single exon of *GPR174* that causes an amino acid substitution [13]. The amino acid substitution in *GPR174* maps to the second extracellular loop region, which is required for ligand recognition and receptor activation, and this mutation may alter these activities [42]. A nucleotide transition (519T>C) in rs3827440 causes serine [TCT]> proline [CCT] [43]. The SNPs rs3810711 and rs3810712 are both located in the 5′ UTR of *GPR174*. The SNP rs5912838, located between the immune receptor *GPR174* and *ITM2A*, was identified as an important signal on Xq21.1 associated with GC in a Chinese Han population [22]. Two SNPs, rs3810711 and rs3810712, both located in the 5′ UTR of *GPR174*, have been reported in perfect LD with rs3827440 [13]. In the present study, we also found rs3827440 to be in perfect LD with rs3810711 and rs3810712 and in strong LD with rs5912838 (Figure 1B).

Our study revealed that rs3827440 TT/T and rs5912838 AA/A were disease-susceptible genotypes and rs3827440 CC/C and rs5912838 CC/C were disease-protective genotypes in the overall AITD, HD, GD, GD-TAO and GD-non-TAO patient groups. When cases and controls were analyzed according to sex, rs3827440 TT and rs5912838 AA were disease-susceptible genotypes and rs3827440 CC and rs5912838 CC showed disease-protective genotypes in female AITD, GD, GD-TAO, and GD-non-TAO patients. In male AITD patients, rs3827440 T and rs5912838 A were disease-susceptible genotypes and rs3827440 C and rs5912838 C were disease-protective genotypes. The T allele of rs3827440 in *GPR174* has been suggested to be GD susceptible in both Chinese and Polish population [22,44]. Chu et al. found that freshly isolated peripheral blood cells from both female homozygous carriers and male carriers of the risk allele T rs3827440 showed a higher level of *GPR174* expression [13]. Ye et al. showed that the transcription level of *ITM2A* in PBMCs from volunteers was regulated by the different alleles of rs3827440 and its linked SNP rs5912838 [45]. Lyso-phosphatidyl-serine acts via *GPR174* and G_s_ α subunit to suppress IL-2 production by activated T cells and limit upregulation of the activation markers CD25 and CD69 [46]. Some studies suggested *GPR174* as an abundantly expressed gas-dependent receptor that can negatively regulate naive T-cell activation [46]. Thus, *GPR174* could mediate an important regulatory pathway connected to central T cell development and peripheral function [47]. In additions, the *GPR174* and *ITM2A* transcripts in CD4 and CD8 T, Natural killer (NK), and monocyte cells with rs3827440 CC or rs5912838 CC genotypes were lower than those with rs3827440 TT or rs5912838 AA genotypes (https://dice-database.org/eqtls). These results might suggest that rs3827440 TT or rs5912838 AA could be more the causative variants in AITD children in males, females and a combination of the sexes.

In genomic linkage and association studies, the X chromosome is less well studied than autosomes, predominantly because of the higher complexity of analyses [47]. Traits and markers on the X chromosome are different from autosomal markers with respect to HWE [48]. The complicating factor for assessing deviation from HWE is that males are hemizygous, and have only one allele on X-chromosomal markers outside of the pseudoautosomal regions, while females have two alleles on the autosomes [49]. Some ignore male subjects and conduct tests for HWE in females only. However, this reduces the sample size and results in a loss of power, and if males are neglected, deviation from HWE cannot be thoroughly investigated [49]. In this present study, we confirmed that the data were in HWE using the calculation proposed by Graffelman and Weir [25]. We specifically analyzed data from cases and controls by sex-stratification or a combination of the sexes to assess the role of *GPR174* and *ITM2A* in immunopathogenesis of female-predominant AITD. To the best of our knowledge, this is the first report of an association between *GPR174* and *ITM2A* SNPs and children with AITD. However, there are limitations of the small sample size, and the controls were not investigated via laboratory methods to exclude subclinical cases of AITD in this study. To confirm the role of *GPR174* and *ITM2A* genes in AITD, further studies on gene expression of immune cells with ethnically diverse populations including large numbers of patients are necessary.

In conclusion, we found that rs3827440 TT/T and rs5912838 AA/A in *GPR174* and *ITM2A* genes on the X chromosome were disease-susceptible genotypes and rs3827440 CC/C and rs5912838 CC/C were disease-protective genotypes among female, male and combined AITD patient groups. In investigating X chromosome data, full use of samples and detailed analysis of data are necessary for case and control disease association genetic studies. These results suggest that polymorphisms in *GPR174* and *ITM2A* genes on the X chromosome play a role in the immunopathogenesis of female-predominant AITD in Korean children.

## Figures and Tables

**Table 1:** Characteristics of 115 autoimmune thyroid disease (AITD) patients and controls.

	AITD (n = 115)		Controls (n = 204)
	GD (n = 74)	HD (n = 41)	
Females (%)	52 (70.3%)	38 (92.7%)	100 (49.0%)
Age at enrollment (years)	14.7 ± 3.6	13.4 ± 3.2	22.7 ± 3.8
Goiter (%)	62 (83.8%)	25 (61.0%)	
T3 at diagnosis, 0.78–1.82 ng/mL	3.79 ± 2.05	1.22 ± 0.55	n.d.
Free T4 at diagnosis, 0.85–1.86 ng/dL	3.17 ± 1.32	1.07 ± 0.74	n.d.
TSH at diagnosis, 0.17–4.05 mIU/L	0.06 ± 0.20	30.57 ± 48.36	n.d.
TSHR Ab positive at diagnosis	72 (97.3%)		n.d.
Tg Ab positive at diagnosis		32 (78%)	n.d.
TPO Ab positive at diagnosis		36 (87.8%)	n.d.
Clinically evident TAO (NOSPECS class II or higher), n (%)	40 (54.1%)		
Remission	23 (31.1%)		

Data are presented as Mean ± SD or n (%), Abbreviations: n.d., not done; AITD, autoimmune thyroid diseases; GD, Graves’ disease; HD, Hashimoto’s disease; TSH, thyroid Stimulating Hormone; TSHR Ab, TSH receptor antibody; TPO, Thyroid Peroxidase; TAO, thyroid associated ophthalmopathy; Remission, consistent with the improvement of clinical features and restoration of euthyroidism or induction of hypothyroidism after ATD therapy [23,24,25].

**Table 2:** Oligonucleotide sequences of primers for multiplex PCR amplifications.

	Gene	RS Number (SNP)	SNP Position (hg19)	Direction	Sequence (5′-3′)	Span ^a^	Specific		
							Tm	Insert Length	Amplicon Size (bp)
PCR1 primer	GPR174	rs3810712	78,426,471	Forward	ACACTCTTTCCCTACACGACGCTCTTCCGATCT **TTG GAA GGA ACA GCA GTT GAT TG**	34	63	65	95
				Reverse	GTGACTGGAGTTCAGACGTGTGCTCTTCCGATCT **ACG TGT AAT TAG CAG GCA TGA TTC TCT CTA**			65	
	GPR174	rs3810711	78,426,488	Forward	ACACTCTTTCCCTACACGACGCTCTTCCGATCT **TTG GAA GGA ACA GCA GTT GAT TGT GAA TTT A**	34		65	95
				Reverse	GTGACTGGAGTTCAGACGTGTGCTCTTCCGATCT **ACG TGT AAT TAG CAG GCA TGA TTC TCT CTA**			65	
	GPR174	rs3827440	78,426,988	Forward	ACACTCTTTCCCTACACGACGCTCTTCCGATCT **CCT GTG TAC TCT TTC CAC TCC TCA GAA**	39		61	97
				Reverse	GTGACTGGAGTTCAGACGTGTGCTCTTCCGATCT **GCC AGG TTG ACA TTC CTG GTA GGA AGA TCC A**			66	
	ITM2A-GPR174	rs5912838	78,497,118	Forward	ACACTCTTTCCCTACACGACGCTCTTCCGATCT **TTC CAC TTC ATG TTA GAT AAA TTT GGA TGT CA**	25		66	86
				Reverse	GTGACTGGAGTTCAGACGTGTGCTCTTCCGATCT **ACT ATG ATC ACA TTT CTC TGG ATA CTT GA**			64	
PCR2 Illumina adapters (sequencing)				MiSeq_F	AATGATACGGCGACCACCGAGATCTACAC	-	59	-	-
				MiSeq_R	CAAGCAGAAGACGGCATACGAGAT				

Bold-face font indicates PCR1 primer region. Underlined regions indicate universal forward and reverse primer tags sequence. The universal primer consists of tag, index and adapter sequences. Between the tag sequence and the Illumina adapter sequence exists an index sequence to specify each sample. Index sequences used in this study (5′-3′): forward (TATAGCCT, ATAGAGGC, CCTATCCT, G GCTCTGA, AGGCGAAG, TAATCTTA, CAGGACGT, GTACTGAC) and reverse (ATTACTCG, TCCGGAGA, CGCTCATT, GAGATTCC, ATTCAGAA, GAATTCGT); ^a^ span: the distance between forward and reverse primers on target sequence.

**Table 3:** Genetic influence of *GPR174* and *ITM2A* SNPs on X chromosome in AITD patients.

			Controls		AITD (GD and HD) *							
	Analysis Type		n = 204 (%)		n = 115 (%)		χ2	*p*-Value	Pc	OR	95CI (Low)	95CI (High)
			F100,	M104	F90,	M25						
GPR174 rs3810712 C>T	F genotype	CC	28	(28.0)	39	(43.3)	4.879	0.027	0.082	2.0	1.1	3.6
		CT	48	(48.0)	41	(45.6)	0.114	0.736	2.208	NA	NA	NA
		TT	24	(24.0)	10	(11.1)	5.356	0.021	0.062	0.4	0.2	0.9
	Comb Genotype	CC/C	66	(32.4)	55	(47.8)	7.479	0.006	0.019	1.9	1.2	3.1
		CT	48	(23.5)	41	(35.7)	5.373	0.020	0.061	1.8	1.1	3.0
		TT/T	87	(42.6)	19	(16.5)	22.623	0.000	0.000	0.3	0.2	0.5

	F Allele	C	104	(52.0)	119	(66.1)	7.780	0.005	0.011	1.8	1.2	2.7
		T	96	(48.0)	61	(33.9)	7.780	0.005	0.011	0.6	0.4	0.8
	M Allele	C	41	(39.4)	16	(64.0)	4.936	0.026	0.053	2.7	1.1	6.8
		T	66	(63.5)	9	(36.0)	6.245	0.012	0.025	0.3	0.1	0.8
	Comb Allele	C	145	(47.7)	135	(65.9)	16.308	0.000	0.000	2.1	1.5	3.0
		T	162	(53.3)	70	(34.1)	18.089	0.000	0.000	0.5	0.3	0.7
GPR174 rs3810711 T>C	F genotype	TT	28	(28.0)	39	(43.3)	4.879	0.027	0.082	2.0	1.1	3.6
		TC	48	(48.0)	41	(45.6)	0.114	0.736	2.208	NA	NA	NA
		CC	24	(24.0)	10	(11.1)	5.356	0.021	0.062	0.4	0.2	0.9
	Comb Genotype	TT/T	66	(32.4)	55	(47.8)	7.479	0.006	0.019	1.9	1.2	3.1
		TC	48	(23.5)	41	(35.7)	5.373	0.020	0.061	1.8	1.1	3.0
		CC/C	87	(42.6)	19	(16.5)	22.623	0.000	0.000	0.3	0.2	0.5
	F Allele	T	104	(52.0)	119	(66.1)	7.780	0.005	0.011	1.8	1.2	2.7
		C	96	(48.0)	61	(33.9)	7.780	0.005	0.011	0.6	0.4	0.8
	M Allele	T	41	(39.4)	16	(64.0)	4.936	0.026	0.053	2.7	1.1	6.8
		C	66	(63.5)	9	(36.0)	6.245	0.012	0.025	0.3	0.1	0.8
	Comb Allele	T	145	(47.7)	135	(65.9)	16.308	0.000	0.000	2.1	1.5	3.0
		C	162	(53.3)	70	(34.1)	18.089	0.000	0.000	0.5	0.3	0.7
GPR174 rs3827440 T>C	F genotype	TT	28	(28.0)	39	(43.3)	4.879	0.027	0.082	2.0	1.1	3.6
		TC	48	(48.0)	41	(45.6)	0.114	0.736	2.208	NA	NA	NA
		CC	24	(24.0)	10	(11.1)	5.356	0.021	0.062	0.4	0.2	0.9
	Comb Genotype	TT/T	66	(32.4)	55	(47.8)	7.479	0.006	0.019	1.9	1.2	3.1
		TC	48	(23.5)	41	(35.7)	5.373	0.020	0.061	1.8	1.1	3.0
		CC/C	87	(42.6)	19	(16.5)	22.623	0.000	0.000	0.3	0.2	0.5
	F Allele	T	104	(52.0)	119	(66.1)	7.780	0.005	0.011	1.8	1.2	2.7
		C	96	(48.0)	61	(33.9)	7.780	0.005	0.011	0.6	0.4	0.8
	M Allele	T	41	(39.4)	16	(64.0)	4.936	0.026	0.053	2.7	1.1	6.8
		C	66	(63.5)	9	(36.0)	6.245	0.012	0.025	0.3	0.1	0.8
	Comb Allele	T	145	(47.7)	135	(65.9)	16.308	0.000	0.000	2.1	1.5	3.0
		C	162	(53.3)	70	(34.1)	18.089	0.000	0.000	0.5	0.3	0.7
ITM2A-GPR174 rs5912838 A>C	F genotype	AA	29	(29.0)	39	(43.3)	4.235	0.040	0.119	1.9	1.0	3.4
		AC	44	(44.0)	41	(45.6)	0.046	0.830	2.489	NA	NA	NA
		CC	27	(27.0)	10	(11.1)	7.626	0.006	0.017	0.3	0.2	0.7
	Comb Genotype	AA/A	68	(33.3)	55	(47.8)	6.520	0.011	0.032	1.8	1.1	2.9
		AC	44	(21.6)	41	(35.7)	7.463	0.006	0.019	2.0	1.2	3.3
		CC/C	89	(43.6)	19	(16.5)	24.129	0.000	0.000	0.3	0.1	0.4
	F Allele	A	102	(51.0)	119	(66.1)	8.890	0.003	0.006	1.9	1.2	2.8
		C	98	(49.0)	61	(33.9)	8.890	0.003	0.006	0.5	0.4	0.8
	M Allele	A	42	(40.4)	16	(64.0)	4.542	0.033	0.066	2.6	1.1	6.5
		C	65	(62.5)	9	(36.0)	5.787	0.016	0.032	0.34	0.1	0.8
	Comb Allele	A	144	(47.4)	135	(65.9)	16.891	0.000	0.000	2.1	1.5	3.1
		C	163	(53.6)	70	(34.1)	18.703	0.000	0.000	0.4	0.3	0.6

GD, Grave’s disease; F, female; Comb, female and male; M, male; * Male allele analysis, which can be counted by heterozygous allele as diploids; χ2, Chi squares; P_c_, Bonferroni’s correction; NA, not applicable; OR, odds ratio.; Total (number of reads supporting one of the four bases above) cut off>=100, MAF (frequency of minor allele)>=0.25.; The value limited all Corrected P (Pc) < 0.005, and *p* < 0.05.

**Table 4:** Genotype(2n) influence of *GPR174* and *ITM2A* SNPs on X chromosome in GD patients with or without TAO.

			Controls		GD								GD_TAO								GD_w/o TAO							
	Genotype Analysis		Total n = 204		Total n = 74		χ2	*p*-Value	Pc	OR	95CI (Low)	95CI (High)	Total n = 40		χ2	*p*-Value	Pc	OR	95CI (Low)	95CI (High)	Total n = 34		χ2	*p*-Value	Pc	OR	95CI (Low)	95CI (High)
			F100 M104 (%)		F52 M22 (%)								F27 M13 (%)								F25 M9 (%)							
**GPR174** **rs3810712** **C>T**	**F**	**CC**	28	(28.0)	26	(50.0)	7.229	0.007	0.022	2.6	1.3	5.2	12	(44.4)	2.665	0.103	0.308	NA	NA	NA	14	(56.0)	7.028	0.008	0.024	3.3	1.3	8.1
		**CT**	48	(48.0)	24	(46.2)	0.047	0.829	2.486	NA	NA	NA	14	(51.9)	0.126	0.722	2.167	NA	NA	NA	10	(40.0)	0.515	0.473	1.419	NA	NA	NA
		**TT**	24	(24.0)	2	(3.8)	9.800	0.002	0.005	0.1	0.0	0.6	1	(3.7)	5.539	0.019	0.056	0.1	0.0	0.9	1	(4.0)	5.000	0.025	0.076	0.1	0.0	1.0
	**Comb**	**CC/C**	66	(32.4)	39	(52.7)	9.567	0.002	0.006	2.3	1.4	4.0	19	(47.5)	3.380	0.066	0.198	NA	NA	NA	20	(58.8)	8.849	0.003	0.009	3.0	1.4	6.3
		**TC**	48	(23.5)	24	(32.4)	0.047	0.829	2.486	NA	NA	NA	14	(35.0)	0.126	0.722	2.167	NA	NA	NA	10	(29.4)	0.515	0.473	1.419	NA	NA	NA
		**TT/T**	87	(42.6)	11	(14.9)	18.841	0.000	0.000	0.2	0.1	0.5	7	(17.5)	8.930	0.003	0.008	0.3	0.1	0.7	4	(11.8)	11.769	0.001	0.002	0.2	0.1	0.5
**GPR174** **rs3810711** **T>C**	**F**	**TT**	28	(28.0)	26	(50.0)	7.229	0.007	0.022	2.6	1.3	5.2	12	(44.4)	2.665	0.103	0.308	NA	NA	NA	14	(56.0)	7.028	0.008	0.024	3.3	1.3	8.1
		**TC**	48	(48.0)	24	(46.2)	0.047	0.829	2.486	NA	NA	NA	14	(51.9)	0.126	0.722	2.167	NA	NA	NA	10	(40.0)	0.515	0.473	1.419	NA	NA	NA
		**CC**	24	(24.0)	2	(3.8)	9.800	0.002	0.005	0.1	0.0	0.6	1	(3.7)	5.539	0.019	0.056	0.1	0.0	0.9	1	(4.0)	5.000	0.025	0.076	0.1	0.0	1.0
	**Comb**	**TT/T**	66	(32.4)	39	(52.7)	9.567	0.002	0.006	2.3	1.4	4.0	19	(47.5)	3.380	0.066	0.198	NA	NA	NA	20	(58.8)	8.849	0.003	0.009	3.0	1.4	6.3
		**TC**	48	(23.5)	24	(32.4)	0.047	0.829	2.486	NA	NA	NA	14	(35.0)	0.126	0.722	2.167	NA	NA	NA	10	(29.4)	0.515	0.473	1.419	NA	NA	NA
		**CC/C**	87	(42.6)	11	(14.9)	18.841	0.000	0.000	0.2	0.1	0.5	7	(17.5)	8.930	0.003	0.008	0.3	0.1	0.7	4	(11.8)	11.769	0.001	0.002	0.2	0.1	0.5
**GPR174** **rs3827440** **T>C**	**F**	**TT**	28	(28.0)	26	(50.0)	7.229	0.007	0.022	2.6	1.3	5.2	12	(44.4)	2.665	0.103	0.308	NA	NA	NA	14	(56.0)	7.028	0.008	0.024	3.3	1.3	8.1
		**TC**	48	(48.0)	24	(46.2)	0.047	0.829	2.486	NA	NA	NA	14	(51.9)	0.126	0.722	2.167	NA	NA	NA	10	(40.0)	0.515	0.473	1.419	NA	NA	NA
		**CC**	24	(24.0)	2	(3.8)	9.800	0.002	0.005	0.1	0.0	0.6	1	(3.7)	5.539	0.019	0.056	0.1	0.0	0.9	1	(4.0)	5.000	0.025	0.076	0.1	0.0	1.0
	**Comb**	**TT/T**	66	(32.4)	39	(52.7)	9.567	0.002	0.006	2.3	1.4	4.0	19	(47.5)	3.380	0.066	0.198	NA	NA	NA	20	(58.8)	8.849	0.003	0.009	3.0	1.4	6.3
		**TC**	48	(23.5)	24	(32.4)	0.047	0.829	2.486	NA	NA	NA	14	(35.0)	0.126	0.722	2.167	NA	NA	NA	10	(29.4)	0.515	0.473	1.419	NA	NA	NA
		**CC/C**	87	(42.6)	11	(14.9)	18.841	0.000	0.000	0.2	0.1	0.5	7	(17.5)	8.930	0.003	0.008	0.3	0.1	0.7	4	(11.8)	11.769	0.001	0.002	0.2	0.1	0.5
**ITM2A-GPR174** **rs5912838** **A>C**	**F**	**AA**	29	(29.0)	26	(50.0)	6.534	0.011	0.032	2.4	1.2	4.9	12	(44.4	2.320	0.128	0.383	NA	NA	NA	14	(56.0)	6.461	0.011	0.033	3.1	1.3	7.7
		**AC**	44	(44.0)	24	(46.2)	0.064	0.800	2.400	NA	NA	NA	14	(51.9	0.528	0.467	1.402	NA	NA	NA	10	(40.0)	0.130	0.718	2.154	NA	NA	NA
		**CC**	27	(27.0)	2	(3.8)	11.879	0.001	0.002	0.1	0.0	0.5	1	(3.7)	6.713	0.010	0.029	0.1	0.0	0.8	1	(4.0)	6.087	0.014	0.041	0.1	0.0	0.9
	**Comb**	**AA/A**	68	(33.3)	39	(52.7)	8.605	0.003	0.010	2.2	1.3	3.8	19	(47.5)	2.925	0.087	0.262	NA	NA	NA	20	(58.8)	8.126	0.004	0.013	2.9	1.4	6.0
		**AC**	44	(21.6)	24	(32.4)	0.064	0.800	2.400	NA	NA	NA	14	(35.0)	0.528	0.467	1.402	NA	NA	NA	10	(29.4)	0.130	0.718	2.154	NA	NA	NA
		**CC/C**	89	(43.6)	11	(14.9)	19.505	0.000	0.000	0.2	0.1	0.5	7	(17.5)	9.566	0.002	0.006	0.3	0.1	0.6	4	(11.8)	12.428	0.000	0.001	0.2	0.1	0.5

GD, Grave’s disease; TAO, thyroid-associated ophthalmopathy; F, female; Comb, female and male; M, male; χ2, Chi squares; P_c_, Bonferroni’s correction; NA, not applicable; OR, odds ratio; w/o, without; CI, confidential interval; total (number of reads supporting one of the four bases above) cut off>=100, MAF (frequency of minor allele) >= 0.25. The value limited all Pc < 0.005, and *p* < 0.05.

**Table 5:** Allele(n) influence of *GPR174* and *ITM2A* SNPs on X chromosome in GD patients with or without TAO.

			Controls		GD								GD_TAO								GD_w/o TAO							
			Total n = 204		Total n = 74		χ2	*p*-Value	Pc	OR	95CI (Low)	95CI (High)	Total n = 40		χ2	*p*-Value	Pc	OR	95CI (Low)	95CI (High)	Total n = 34		χ2	*p*-Value	Pc	OR	95CI (Low)	95CI (High)
Allele			F100 M104 (%)		F52 M22 (%)								F27 M13 (%)								F25 M9 (%)							
**GPR174** **rs3810712** **C>T**	**F**	**C**	104	(52.0)	76	(73.1)	12.585	0.000	0.001	2.5	1.5	4.2	38	(70.4)	5.821	0.016	0.032	2.2	1.1	4.2	38	(76.0)	9.390	0.002	0.004	2.9	1.4	5.9
		**T**	96	(48.0)	28	(26.9)	12.585	0.000	0.001	0.4	0.2	0.7	16	(29.6)	5.821	0.016	0.032	0.5	0.2	0.9	12	(24.0)	9.390	0.002	0.004	0.3	0.2	0.7
	**M**	**C**	41	(39.4)	13	(59.1)	2.868	0.090	0.181	NA	NA	NA	7	(53.8)	0.994	0.319	0.638	NA	NA	NA	6	(66.7)	2.531	0.112	0.223	NA	NA	NA
		**T**	66	(63.5)	9	(40.9)	3.833	0.050	0.100	NA	NA	NA	6	(46.2)	1.463	0.227	0.453	NA	NA	NA	3	(33.3)	3.162	0.075	0.151	NA	NA	NA
	**Comb**	**C**	145	(47.7)	89	(70.6)	18.894	0.000	0.000	2.6	1.7	4.1	45	(67.2)	8.327	0.004	0.008	2.2	1.3	3.9	44	(74.6)	14.304	0.000	0.000	3.2	1.7	6.0
		**T**	162	(53.3)	37	(29.4)	20.508	0.000	0.000	0.4	0.2	0.6	22	(32.8)	9.188	0.002	0.005	0.4	0.2	0.7	15	(25.4)	15.356	0.000	0.000	0.3	0.2	0.6
**GPR174** **rs3810711** **T>C**	**F**	**T**	104	(52.0)	76	(73.1)	12.585	0.000	0.001	2.5	1.5	4.2	38	(70.4)	5.821	0.016	0.032	2.2	1.1	4.2	38	(76.0)	9.390	0.002	0.004	2.9	1.4	5.9
		**C**	96	(48.0)	28	(26.9)	12.585	0.000	0.001	0.4	0.2	0.7	16	(29.6)	5.821	0.016	0.032	0.5	0.2	0.9	12	(24.0)	9.390	0.002	0.004	0.3	0.2	0.7
	**M**	**T**	41	(39.4)	13	(59.1)	2.868	0.090	0.181	NA	NA	NA	7	(53.8)	0.994	0.319	0.638	NA	NA	NA	6	(66.7)	2.531	0.112	0.223	NA	NA	NA
		**C**	66	(63.5)	9	(40.9)	3.833	0.050	0.100	NA	NA	NA	6	(46.2)	1.463	0.227	0.453	NA	NA	NA	3	(33.3)	3.162	0.075	0.151	NA	NA	NA
	**Comb**	**T**	145	(47.7)	89	(70.6)	18.894	0.000	0.000	2.6	1.7	4.1	45	(67.2)	8.327	0.004	0.008	2.2	1.3	3.9	44	(74.6)	14.304	0.000	0.000	3.2	1.7	6.0
		**C**	162	(53.3)	37	(29.4)	20.508	0.000	0.000	0.4	0.2	0.6	22	(32.8)	9.188	0.002	0.005	0.4	0.2	0.7	15	(25.4)	15.356	0.000	0.000	0.3	0.2	0.6
**GPR174** **rs3827440** **T>C**	**F**	**T**	104	(52.0)	76	(73.1)	12.585	0.000	0.001	2.5	1.5	4.2	38	(70.4)	5.821	0.016	0.032	2.2	1.1	4.2	38	(76.0)	9.390	0.002	0.004	2.9	1.4	5.9
		**C**	96	(48.0)	28	(26.9)	12.585	0.000	0.001	0.4	0.2	0.7	16	(29.6)	5.821	0.016	0.032	0.5	0.2	0.9	12	(24.0)	9.390	0.002	0.004	0.3	0.2	0.7
	**M**	**T**	41	(39.4)	13	(59.1)	2.868	0.090	0.181	NA	NA	NA	7	(53.8)	0.994	0.319	0.638	NA	NA	NA	6	(66.7)	2.531	0.112	0.223	NA	NA	NA
		**C**	66	(63.5)	9	(40.9)	3.833	0.050	0.100	NA	NA	NA	6	(46.2)	1.463	0.227	0.453	NA	NA	NA	3	(33.3)	3.162	0.075	0.151	NA	NA	NA
	**Comb**	**T**	145	(47.7)	89	(70.6)	18.894	0.000	0.000	2.6	1.7	4.1	45	(67.2)	8.327	0.004	0.008	2.2	1.3	3.9	44	(74.6)	14.304	0.000	0.000	3.2	1.7	6.0
		**C**	162	(53.3)	37	(29.4)	20.508	0.000	0.000	0.4	0.2	0.6	22	(32.8)	9.188	0.002	0.005	0.4	0.2	0.7	15	(25.4)	15.356	0.000	0.000	0.3	0.2	0.6
**ITM2A-GPR174** **rs5912838** **A>C**	**F**	**A**	102	(51.0)	76	(73.1)	13.741	0.000	0.000	2.6	1.6	4.4	38	(70.4)	6.449	0.011	0.022	2.3	1.2	4.4	38	(76.0)	10.146	0.001	0.003	3.0	1.5	6.2
		**C**	98	(49.0)	28	(26.9)	13.741	0.000	0.000	0.4	0.2	0.6	16	(29.6)	6.449	0.011	0.022	0.4	0.2	0.8	12	(24.0)	10.146	0.001	0.003	0.3	0.2	0.7
	**M**	**A**	42	(40.4)	13	(59.1)	2.583	0.108	0.216	NA	NA	NA	7	(53.8)	0.860	0.354	0.707	NA	NA	NA	6	(66.7)	2.342	0.126	0.252	NA	NA	NA
		**C**	65	(62.5)	9	(40.9)	3.492	0.062	0.123	NA	NA	NA	6	(46.2)	1.294	0.255	0.511	NA	NA	NA	3	(33.3)	2.940	0.086	0.173	NA	NA	NA
	**Comb**	**A**	144	(47.4)	89	(70.6)	19.425	0.000	0.000	2.7	1.7	4.2	45	(67.2)	8.609	0.003	0.007	2.3	1.3	4.0	44	(74.6)	14.650	0.000	0.000	3.3	1.7	6.1
		**C**	163	(53.6)	37	(29.4)	21.062	0.000	0.000	0.4	0.2	0.6	22	(32.8)	9.485	0.002	0.004	0.4	0.2	0.7	15	(25.4)	15.717	0.000	0.000	0.3	0.2	0.6

GD, Grave’s disease; TAO, thyroid-associated ophthalmopathy; F, female; Comb, female and male; M, male; Male allele analysis, which can be counted by heterozygous allele as diploids; χ2, Chi squares; P_c_, Bonferroni’s correction; NA, not applicable; OR, odds ratio; w/o, without; CI, confidential interval; total (number of reads supporting one of the four bases above) cut off >= 100, MAF (frequency of minor allele) >= 0.25.The value limited all Pc < 0.005, and *p* < 0.05.

**Table 6:** Four *GPR174* and *ITM2A* SNPs haplotype frequencies identified in controls and AITD patents.

Haplotype	Freq.	Case, Control Ratio Counts		Case, Control Frequencies		Chi Square	*p*-Value
		AITD	Control	AITD	Control		
		+ #x2003;-	+#x2003;#x2003;-				
CTTA	0.537	135.0:70.0	137.0:164.0	0.658	0.455	20.29	0.000
TCCC	0.445	70.0:135.0	155.0:146.0	0.341	0.515	14.87	0.000

## Supplementary Materials

The following are available online at https://www.mdpi.com/2073-4425/11/8/858/s1, Table S1: Hardy Weinberg equilibrium for GPR174 four SNPs with or without males, Table S2: Hardy Weinberg equilibrium for GPR174 four SNPs with case/control analysis using Haploview.

## References

[1] Tomer Y., Davies T.F. (2003). Searching for the autoimmune thyroid disease susceptibility genes: from gene mapping to gene function. Endocr. Rev..

[2] Brix T.H., Kyvik K.O., Christensen K., Hegedüs L. (2001). Evidence for a major role of heredity in Graves’ disease: a population-based study of two Danish twin cohorts. J. Clin. Endocrinol. Metab..

[3] Stassi G., De Maria R. (2002). Autoimmune thyroid disease: new models of cell death in autoimmunity. Nat. Rev. Immunol..

[4] Huh K.B., Lee H.C., Kim H.M., Lee H.R., Hong C.S., Lee S.Y., Choi H.J., Park K., Kim C.K. (1986). Human leukocyte antigen (HLA) in Korean patients with autoimmune thyroid diseases. Korean J. Int. Med..

[5] Honda K., Tamai H., Morita T., Kuma K., Nishimura Y., Sasazuki T. (1989). Hashimoto’s thyroiditis and HLA in Japanese. J. Clin. Endocrinol. Metab..

[6] Webb R., Kelly J.A., Somers E.C., Hughes T., Kaufman K.M., Sanchez E., Nath S.K., Bruner G., Alarcon-Riquelme M.E., Gilkeson G.S. (2011). Early disease onset is predicted by a higher genetic risk for lupus and is associated with a more severe phenotype in lupus patients. Ann. Rheumatic Diseases.

[7] Cho W.K., Jung M.H., Choi E.J., Choi H.B., Kim T.G., Suh B.K. (2011). Association of HLA alleles with autoimmune thyroid disease in Korean children. Horm. Res. Paediatr..

[8] Cho B.Y., Rhee B.D., Lee D.S., Lee M.S., Kim G.Y., Lee H.K., Koh C.S., Min H.K., Lee M. (1987). HLA and Graves’ disease in Koreans. Tissue Antigens.

[9] Libert C., Dejager L., Pinheiro I. (2010). The X chromosome in immune functions: when a chromosome makes the difference. Nat. Rev. Immunol..

[10] Whitacre C.C. (2001). Sex differences in autoimmune disease. Nat. Immunol..

[11] Dai R., Ahmed S.A. (2014). Sexual dimorphism of miRNA expression: a new perspective in understanding the sex bias of autoimmune diseases. Therapeutics Clin. Risk Manag..

[12] Fish E.N. (2008). The X-files in immunity: sex-based differences predispose immune responses. Nat. Rev. Immunol..

[13] Chu X., Shen M., Xie F., Miao X.J., Shou W.H., Liu L., Yang P.P., Bai Y.N., Zhang K.Y., Yang L. (2013). An X chromosome-wide association analysis identifies variants in GPR174 as a risk factor for Graves’ disease. J. Med. Genetics.

[14] Yin X., Latif R., Tomer Y., Davies T.F. (2007). Thyroid epigenetics: X chromosome inactivation in patients with autoimmune thyroid disease. Ann. N. Y. Acad. Sci..

[15] Chabchoub G., Uz E., Maalej A., Mustafa C.A., Rebai A., Mnif M., Bahloul Z., Farid N.R., Ozcelik T., Ayadi H. (2009). Analysis of skewed X-chromosome inactivation in females with rheumatoid arthritis and autoimmune thyroid diseases. Arthritis Res. Therapy.

[16] Ishido N., Inoue N., Watanabe M., Hidaka Y., Iwatani Y. (2015). The relationship between skewed X chromosome inactivation and the prognosis of Graves’ and Hashimoto’s diseases. Thyroid Off. J. Am. Thyroid Assoc..

[17] Ban Y., Tozaki T., Tobe T., Ban Y., Jacobson E.M., Concepcion E.S., Tomer Y. (2007). The regulatory T cell gene FOXP3 and genetic susceptibility to thyroid autoimmunity: an association analysis in Caucasian and Japanese cohorts. J. Autoimmun..

[18] Inoue N., Watanabe M., Morita M., Tomizawa R., Akamizu T., Tatsumi K., Hidaka Y., Iwatani Y. (2010). Association of functional polymorphisms related to the transcriptional level of FOXP3 with prognosis of autoimmune thyroid diseases. Clin. Exp. Immunol..

[19] Xiao W., Liu Z., Lin J., Li J., Wu K., Ma Y., Xiong C., Gong Y., Liu Z. (2015). Association of Toll-like receptor 7 and 8 gene polymorphisms with Graves’ disease in Chinese Cantonese population. Tissue Antigens.

[20] Song R.H., Qin Q., Yan N., Muhali F.S., Meng S., He S.T., Zhang J.A. (2015). Variants in IRAK1-MECP2 region confer susceptibility to autoimmune thyroid diseases. Mol. Cell. Endocrinol..

[21] Shin H.R., Cho W.K., Baek I.C., Lee N.Y., Lee Y.J., Kim S.K., Ahn M.B., Suh B.K., Kim T.G. (2020). Polymorphisms of IRAK1 gene on X chromosome is associated with Hashimoto’s thyroiditis in Korean Children. Endocrinology.

[22] Zhao S.X., Xue L.Q., Liu W., Gu Z.H., Pan C.M., Yang S.Y., Zhan M., Wang H.N., Liang J., Gao G.Q. (2013). Robust evidence for five new Graves’ disease risk loci from a staged genome-wide association analysis. Hum. Mol. Genetics.

[23] Gastaldi R., Poggi E., Mussa A., Weber G., Vigone M.C., Salerno M., Delvecchio M., Peroni E., Pistorio A., Corrias A. (2014). Graves disease in children: thyroid-stimulating hormone receptor antibodies as remission markers. J. Pediatr..

[24] Inoue N., Watanabe M., Morita M., Tatusmi K., Hidaka Y., Akamizu T., Iwatani Y. (2011). Association of functional polymorphisms in promoter regions of IL5, IL6 and IL13 genes with development and prognosis of autoimmune thyroid diseases. Clin. Exp. Immunol..

[25] Graffelman J., Weir B.S. (2016). Testing for Hardy-Weinberg equilibrium at biallelic genetic markers on the X chromosome. Heredity.

[26] Fisher D.A., Oddie T.H., Johnson D.E., Nelson J.C. (1975). The diagnosis of Hashimoto’s thyroiditis. J. Clin. Endocrinol. Metab..

[27] Werner S.C. (1977). Modification of the classification of the eye changes of Graves’ disease: recommendations of the Ad Hoc Committee of the American Thyroid Association. J. Clin. Endocrinol. Metab..

[28] Frecker M., Stenszky V., Balazs C., Kozma L., Kraszits E., Farid N.R. (1986). Genetic factors in Graves’ ophthalmopathy. Clin. Endocrinol..

[29] Shin D.H., Baek I.C., Kim H.J., Choi E.J., Ahn M., Jung M.H., Suh B.K., Cho W.K., Kim T.G. (2019). HLA alleles, especially amino-acid signatures of HLA-DPB1, might contribute to the molecular pathogenesis of early-onset autoimmune thyroid disease. PLoS ONE.

[30] Schirmer M., Ijaz U.Z., D’Amore R., Hall N., Sloan W.T., Quince C. (2015). Insight into biases and sequencing errors for amplicon sequencing with the Illumina MiSeq platform. Nucleic Acids Res..

[31] Jäger A.C., Alvarez M.L., Davis C.P., Guzmán E., Han Y., Way L., Walichiewicz P., Silva D., Pham N., Caves G. (2017). Developmental validation of the MiSeq FGx Forensic Genomics System for Targeted Next Generation Sequencing in Forensic DNA Casework and Database Laboratories. Forensic. Sci. Int. Genet..

[32] Takahashi S., Tomita J., Nishioka K., Hisada T., Nishijima M. (2014). Development of a prokaryotic universal primer for simultaneous analysis of Bacteria and Archaea using next-generation sequencing. PLoS ONE.

[33] Ravi R.K., Walton K., Khosroheidari M. (2018). MiSeq: A Next Generation Sequencing Platform for Genomic Analysis. Methods Mol. Biol. (Clifton, N.J.).

[34] Sikkema-Raddatz B., Johansson L.F., de Boer E.N., Almomani R., Boven L.G., van den Berg M.P., van Spaendonck-Zwarts K.Y., van Tintelen J.P., Sijmons R.H., Jongbloed J.D. (2013). Targeted next-generation sequencing can replace Sanger sequencing in clinical diagnostics. Hum. Mutat..

[35] Robinson J.T., Thorvaldsdottir H., Winckler W., Guttman M., Lander E.S., Getz G., Mesirov J.P. (2011). Integrative genomics viewer. Nat. Biotechnol..

[36] Li H., Durbin R. (2009). Fast and accurate short read alignment with Burrows-Wheeler transform. Bioinformatics.

[37] Blankenberg D., Von Kuster G., Bouvier E., Baker D., Afgan E., Stoler N., Taylor J., Nekrutenko A. (2014). Dissemination of scientific software with Galaxy ToolShed. Genome Biol..

[38] Rebolledo-Jaramillo B., Su M.S., Stoler N., McElhoe J.A., Dickins B., Blankenberg D., Korneliussen T.S., Chiaromonte F., Nielsen R., Holland M.M. (2014). Maternal age effect and severe germ-line bottleneck in the inheritance of human mitochondrial DNA. Proc. Natl. Acad. Sci. USA.

[39] Jun J.K., Kim S.M. (2012). Association study of fibroblast growth factor 2 and fibroblast growth factor receptors gene polymorphism in korean ossification of the posterior longitudinal ligament patients. J. Korean Neurosurg. Soc..

[40] Waldenlind K., Saevarsdottir S., Bengtsson C., Askling J. (2018). Risk of Thyroxine-Treated Autoimmune Thyroid Disease Associated With Disease Onset in Patients With Rheumatoid Arthritis. JAMA Netw. Open.

[41] Kirchner J., Bevan M.J. (1999). ITM2A is induced during thymocyte selection and T cell activation and causes downregulation of CD8 when overexpressed in CD4(+)CD8(+) double positive thymocytes. J. Exp. Med..

[42] Peeters M.C., van Westen G.J., Li Q., AP I.J. (2011). Importance of the extracellular loops in G protein-coupled receptors for ligand recognition and receptor activation. Trends Pharmacol. Sci..

[43] https://www.ncbi.nlm.nih.gov/snp/rs3827440.

[44] Szymanski K., Miskiewicz P., Pirko K., Jurecka-Lubieniecka B., Kula D., Hasse-Lazar K., Krajewski P., Bednarczuk T., Ploski R. (2014). rs3827440, a nonsynonymous single nucleotide polymorphism within GPR174 gene in X chromosome, is associated with Graves’ disease in Polish Caucasian population. Tissue Antigens.

[45] Ye X.P., Yuan F.F., Zhang L.L., Ma Y.R., Zhang M.M., Liu W., Sun F., Wu J., Lu M., Xue L.Q. (2017). ITM2A Expands Evidence for Genetic and Environmental Interaction in Graves Disease Pathogenesis. J. Clin. Endocrinol. Metab..

[46] Barnes M.J., Cyster J.G. (2018). Lysophosphatidylserine suppression of T-cell activation via GPR174 requires Gαs proteins. Immunol. Cell Biol..

[47] Napier C., Mitchell A.L., Gan E., Wilson I., Pearce S.H. (2015). Role of the X-linked gene GPR174 in autoimmune Addison’s disease. J. Clin. Endocrinol. Metab..

[48] Puig X., Ginebra J., Graffelman J. (2017). A Bayesian test for Hardy-Weinberg equilibrium of biallelic X-chromosomal markers. Heredity.

[49] Wellek S., Ziegler A. (2019). Testing for goodness rather than lack of fit of an X-chromosomal SNP to the Hardy-Weinberg model. PLoS ONE.

