# Supplementary material for: GPR174 and ITM2A Gene Polymorphisms rs3827440 and rs5912838 on the X chromosome in Korean Children with Autoimmune Thyroid Disease
Source: Genes (Basel). 2020 Jul 27;11(8):858. doi: 10.3390/genes11080858 (PMC7465061; doi:10.3390/genes11080858)
Supplement: Supplementary file 1 [file genes-11-00858-s001.pdf]

Supplementary Table S1. Hardy Weinberg equilibrium for GPR174 four SNPs with or without males

	Genotype and allele					HWp-value	
	mC	mT	fCC	fCT	fTT	ALL	Female
Control	66	39	24	48	28	0.042	0.70
GPR174 (rs3810712 : T>C)	9	16	11	41	38	0.995	0.99
	mT	mC	fTT	fTC	fCC	ALL	Female
Control	39	66	28	48	24	0.042	0.70
GPR174 (rs3810711 : T>C)	16	9	39	41	10	0.966	0.87
	mT	mC	fTT	fTC	fCC	ALL	Female
Control	41	64	28	48	24	0.089	0.70
GPR174 (rs3827440 : T>A/T>C)	16	9	39	41	10	0.966	0.87
	mA	mC	fAA	fAC	fCC	ALL	Female
Control	40	65	29	44	27	0.044	0.23
ITM2A-GPR174 (rs5912838 : A>C)	16	9	39	41	10	0.966	0.87

Supplementary Table S2. Hardy Weinberg equilibrium for GPR174 four SNPs with case/control analysis using Haploview

#	Name	Position	ObsHET	PredHET	HWpval	%Geno	FamTrio	MendErr	MAF	Alleles
1	rs3810712	78,426,471	0.468	0.496	0.7208	99.1	0	0	0.453	C:T
2	rs3810711	78,426,488	0.468	0.496	0.7208	99.1	0	0	0.453	T:C
3	rs3827440	78,426,988	0.468	0.496	0.7208	99.1	0	0	0.453	T:C
4	rs5912838	78,497,118	0.447	0.496	0.3161	99.1	0	0	0.455	A:C

The Chi square test for Hardy-Weinberg (HWE), using two degrees of freedom, was calculated in both sexes according to the following alleles and genotypes: in males, p and q are the number of males carrying respectively allele, while in female, p^2, 2pq and q^2 are the number of females carrying one of the probable respectively genotype. Two times analysis conducted by directly statistical analysis [25] and Haploview software, version 4.2.
